# Integration of Biomass Formulations of Genome-Scale Metabolic Models with Experimental Data Reveals Universally Essential Cofactors in Prokaryotes

**DOI:** 10.1016/j.ymben.2016.12.002

**Published:** 2017-01

**Authors:** Joana C. Xavier, Kiran Raosaheb Patil, Isabel Rocha

**Affiliations:** a CEB - Centre of Biological Engineering, University of Minho, Campus de Gualtar, 4710-057 Braga, Portugal; b Structural and Computational Biology Unit, European Molecular Biology Laboratory, Meyerhofstraße 1, 69117 Heidelberg, Germany

**Keywords:** Essential cofactors, Metabolic networks, Genome-scale models

## Abstract

The composition of a cell in terms of macromolecular building blocks and other organic molecules underlies the metabolic needs and capabilities of a species. Although some core biomass components such as nucleic acids and proteins are evident for most species, the essentiality of the pool of other organic molecules, especially cofactors and prosthetic groups, is yet unclear. Here we integrate biomass compositions from 71 manually curated genome-scale models, 33 large-scale gene essentiality datasets, enzyme-cofactor association data and a vast array of publications, revealing universally essential cofactors for prokaryotic metabolism and also others that are specific for phylogenetic branches or metabolic modes. Our results revise predictions of essential genes in *Klebsiella pneumoniae* and identify missing biosynthetic pathways in models of *Mycobacterium tuberculosis*. This work provides fundamental insights into the essentiality of organic cofactors and has implications for minimal cell studies as well as for modeling genotype-phenotype relations in prokaryotic metabolic networks.

## Highlights

• Seventy one biomass equations of manually curated genome-scale metabolic models are compared.
• Eight classes of universally essential prokaryotic organic cofactors are proposed.
• Conditionally essential organic cofactors are presented and discussed.
• Gene essentiality predictions for *Klebsiella pneumoniae* are revised.
• A missing essential pathway in models of *Mycobacterium tuberculosis* is predicted.

## 1 Introduction

The biomass composition of a cell reflects the genetic repertoire necessary to synthesize, salvage, or uptake the necessary constituents for growth and maintenance. Indeed, it can be used in taxonomical classification (De Ley and Van Muylem, 1963, Hiraishi, 1999, Hoiczyk and Hansel, 2000, Muto and Osawa, 1987, Rosselló-Mora and Amann, 2001, Schleifer and Kandler, 1972) and is intimately related with the species’ growth rates (Bremer and Dennis, 1996, Kemp et al., 1993). Consequently, biomass composition is strongly linked to drug sensitivity, nutritional requirements, and the biosynthetic potential for industrial applications of a species.

There is a considerable lack of standardized protocols (both experimental and computational) and multi-species comparative assays in determining (quantitative and qualitatively) which components make up the cell's biomass, in contrast with the advanced picture in terms of the elemental composition (Whitman et al., 1998). Genome-scale metabolic models (GEMs) have exposed but also underscored the need to reduce this knowledge gap in biomass compositions. GEMs have systematized metabolic knowledge on dozens of microorganisms, with applications in diverse areas, from industrial biotechnology to medical microbiology (Kim et al., 2012, Monk et al., 2014). Biomass composition is a critical element of these models, allowing the representation of cell growth *in silico*. This is performed through a growth reaction wherein necessary constituents are combined in stoichiometric amounts producing new biomass. Maximization of the flux through this reaction, the so-called Biomass Objective Function (BOF), is the most commonly used method for simulating growth phenotypes through the Flux Balance Analysis methodology (Savinell and Palsson, 1992, Varma and Palsson, 1993a).

The BOF can be formulated as a direct biosynthesis from precursor metabolites (Varma and Palsson, 1993a, Varma and Palsson, 1993b); biosynthesis from building blocks (Feist et al., 2007, Varma et al., 1993) or biosynthesis from macromolecules (Liao et al., 2011), using lumped reactions for each (Villadsen et al., 2011). The solute pool or cofactor pool is often added as substrate in one of those reactions (Kim et al., 2010). The macromolecular composition and detailed content of building blocks, together with energetic costs of growth and maintenance, can be sufficient to simulate growth of wild-type organisms (Liao et al., 2011). However, predictions of complex phenotypes, e.g. following gene deletions or in poor media, require the addition of organic and inorganic cofactors and mineral compositions (Feist et al., 2007, Feist and Palsson, 2010). For greater accuracy in predicting reaction and gene essentiality, the BOF should be adapted to include only those components that are strictly essential for the cell – the so called core BOF (Feist and Palsson, 2010, Mendum et al., 2011). However, there is yet no consensus on how certain components as organic cofactors should be included in reactions. For example, Coenzyme A, an important cofactor in lipids metabolism, can be found represented in isolation in the solute pool of the BOF, charged with lipids, or is even excluded from the BOF. These different ways of qualitatively formulating BOFs, together with nomenclature inconsistencies that have been addressed elsewhere (Bernard et al., 2014, Kumar et al., 2012, Sauls and Buescher, 2014), hinder comparative studies involving manually curated GEMs.

The utility of metabolic models is tied to the accuracy of the biomass composition used (Feist et al., 2007, Feist and Palsson, 2010, Mendum et al., 2011). Yet, most GEMs adapt the biomass composition from few well-studied organisms due to the lack of standardized protocols, both experimental and computational. For quantitative analyses of the impact of variations in the stoichiometric coefficients we refer the reader to previous studies (Dikicioglu et al., 2015, Feist et al., 2007, Pramanik and Keasling, 1998, Yuan et al., 2016). Here, we address the qualitative aspect of the problem, specifically pertaining to organic cofactors, by bringing together evidences for essentiality hidden in disparate data sources – biochemical and bioinformatics databases, literature and genetic screens. Organic cofactors, although not consumed in metabolism, are essential for catalysis and need to be distributed in sufficient amounts among the daughter cells (Zhao and van der Donk, 2003). Our analysis reveals several essential organic cofactors for archaeal and bacterial metabolism.

## 2 Results

### 2.1 Universe of biomass constituents in prokaryotic GEMs is large and heterogeneous

**Fig. 1:**
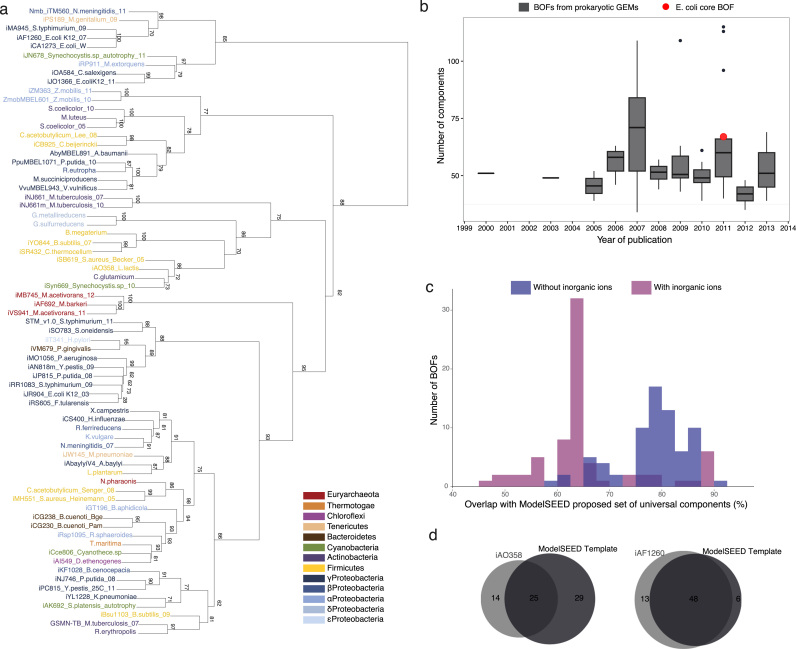
**Comparison of biomass compositions in prokaryotic genome-scale metabolic models. (a)** Cluster dendrogram for qualitative biomass compositions of 71 manually curated GEMs (abbreviations include model ID when available, species name and year and/or first author if more than one model was compared for the same species). Numbers on branches show multi-scale bootstrap resampling probabilities (approximately unbiased p-values, %). **(b)** Qualitative dimension (number of components) of biomass objective functions (BOFs) of manually curated GEMs per year compared with the dimension of the core BOF of *E. coli* published in 2011. **(c)** Distribution of overlaps of the biomass constituents of GEMs with the ModelSEED's proposed set of universal biomass components. In magenta, overlaps including all components; in blue, overlaps excluding inorganic ions from all compared sets. **(d)** Venn diagrams depicting GEMs with smallest and highest overlaps with the ModelSEED template (inorganic ions included), iAO358 (*Lactococcus lactis*) and iAF1260 (*E. coli*) respectively.

We first extensively assessed biomass compositions in published prokaryotic GEMs. In total, 71 detailed biomass compositions were gathered, covering 9 phyla with 5 classes of Proteobacteria and one phylum of Archaea (Supplementary Dataset 1). To enable comparison across different models, we reconciled diverse nomenclatures and representation styles, ranging from lumped stoichiometry to reaction-level inclusion. This exercise resulted in 551 unique metabolites (nomenclature as per BiGG database (Schellenberger et al., 2010)) that are used as biomass constituents, including 20 charged tRNA molecules, 16 inorganic ions and water (Supplementary Datasets 2–4). Of these, more than half – 261 – are present in only one BOF. Clustering of these diverse BOFs revealed large discrepancy between biomass compositions used in models of species in the same phyla (e.g. four species of cyanobacteria) or even between different versions of models of the same species (e.g. *Bacillus subtilis* and *Escherichia coli*), as shown in Fig. 1a. The clustering appears to be affected in some cases by the template biomass composition used in reconstruction, which results in only a few biologically relevant clusters. This is the case of the cluster containing six BOFs of γ-Proteobacteria built based on the BOF of iJR904 (Reed et al., 2003), a model of *E. coli* built in 2003. Another is the cluster that includes all the three models of the genus *Methanosarcina,* built after the first model, iAF692. However, the majority of models group independent of their phylogenetic relatedness. Most of the clusters are thus artifacts rather than representing underlying biology. For example, the node on the bottom of the tree (including *Bacillus subtilis*, *Mycobacterium tuberculosis* and *Rhodococcus erythropolis*) represents the three BOFs with the most unique compounds among all compounds represented in the 71 BOFs analyzed.

The detail of biomass compositions was found not to be correlated with the year of publication, indicating that the emergence of standards was not verified in the period analyzed, and the majority of BOFs have a lower number of components than those indicated as core for *E. coli* in 2011 (Orth et al., 2011) (Fig. 1b). Furthermore, none of the BOFs of the manually curated models included all biomass components deemed universal in the ModelSEED biomass template (Henry et al., 2010) (Fig. 1c). The least comprehensive BOF excludes 29 components and the most comprehensive excludes 6, amidst which are well-known entities such as acyl carrier protein (ACP), AMP and GDP (Fig. 1d). Although the overlap between the BOFs and the ModelSEED template increases considerably when excluding inorganic ions from the analysis, there is still no BOF with 100% overlap (Fig. 1c; Supplementary Datasets 5 and 6).

### 2.2 Qualitative biomass composition drastically impacts essentiality predictions

**Fig. 2:**
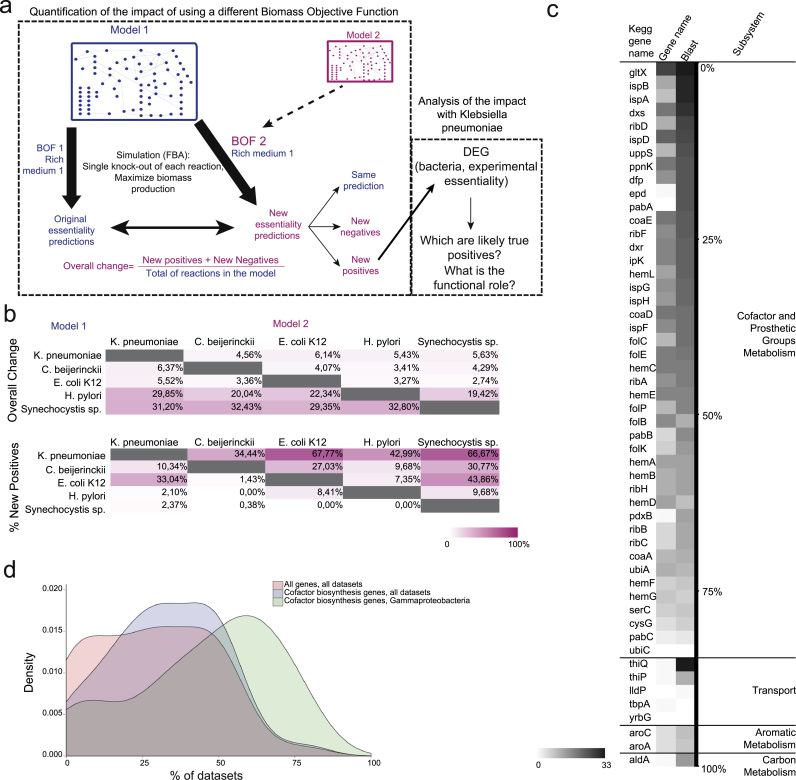
**Impact of biomass composition on predictions of reaction and gene essentiality** (a) Outline of the *in silico* procedure used in the swap study. In blue, data and predictions corresponding to the original model. In magenta, data and predictions with a new BOF (See Section 4.3 for details). (b) Number of reactions changing essentiality status after swapping biomass composition among five GEMs of different prokaryotes. Color scale according to normalized percentages: upper panel – overall change normalized by total of reactions in the model; bottom panel – percentage of new positives in the overall change. (c) Number of mappings – by gene name annotation and protein sequence – of 52 new essential genes predicted for *Klebsiella pneumoniae* (model iYL1228), against all experimentally determined essential genes for 33 bacterial genome-wide essentiality datasets in the database of essential genes (DEG). (d) Percentage of large-scale essentiality datasets in which new essential genes for *K. pneumoniae* show up as essential (density per number of genes). In orange, presence of all new essential genes in the whole DEG database; in light-blue, the subset of new essential genes annotated as involved in cofactor metabolism against all essentiality datasets; in green, new essential genes annotated as involved in cofactor metabolism against datasets of Gammaproteobacteria only.

To assess the impact of the qualitative composition of BOFs on gene and reaction essentiality predictions, we selected five GEMs representing phylogenetically diverse species. Flux Balance Analysis (FBA) (Savinell and Palsson, 1992) was used to predict single reaction essentiality. Then, for each model, the simulations were repeated after swapping the original BOFs with those from the other four models (Fig. 2a; Supplementary Dataset 7). In cases where no growth was observed after the swapping, individual components of the new BOF were removed until growth was observed. These are listed in Supplementary Dataset 8 and consist majorly of lipids, some which are detailed in some models, as *E. coli*'s and *Klebsiella pneumoniae* (both with three phosphoethanolamines named _pe160, _pe161, _pe181) some which are represented in a high level form, tagged with the species’ initials (phosphoethanolamine is named pe_CB in *C. beijerinckii* and pe_HP in *H. pylori*). We also identified gaps, for example in the network of *C. beijerinckii* regarding the production of Spermine, Spermidine, Glutathione, Dimethylbenzimidazole and Adenosylcobalamin. Even under the rich media conditions used (see Section 4.3), wherein the number of essential reactions would be the smallest, we observed considerable changes in essentiality predictions. The impact varied from 2.74% to 32.8% of the reactions changing status from essential to non-essential or vice-versa (Fig. 2b) attesting the fundamental role of biomass composition in the applicability of GEMs.

To gain further insight into the biomass-dependency of essentiality predictions, we classified the altered predictions as new negatives or new positives (See Section 4.3). In the case of *Synechocystis sp*., between 29.4% and 32.8% of essential reactions were different when using an alternative biomass composition (Fig. 2b). Most of these new predictions, however, (from 97.6% to 100%) were new negatives, due to several components including those essential for photosynthesis being removed with the swap (Fig. 2b; Supplementary Dataset 9). Interestingly, in some swaps, new essential reactions were a larger proportion of the overall change. The extreme case was that of iYL1228 (*Klebsiella pneumoniae)* with the BOF of iAF1260 (*E. coli*), wherein 82 (67.7%) of the predictions were new essentials. The BOF of iAF1260 brings 19 new components that iYL1228 can produce (Supplementary Dataset 7; Fig. 2b); no alterations had to be done to the BOF of iAF1260 in order to get iYL1128 to grow (Supplementary Dataset 8). Both species are closely related, belonging to Enterobacteriaceae, a common family of Gammaproteobacteria that includes known pathogens causing concerns due to multidrug-resistance (Pitout and Laupland, 2008), which indicates that the biomass compositions of the two species might be similar and hints at possible gaps in the BOF of iYL1228.

### 2.3 Newly predicted essential genes have essential orthologs in multiple species and are related with cofactor metabolism

To investigate the essentiality and the biological role of the predicted new essential genes of iYL1228, given that there is no large-scale experimental assay of gene essentiality for *K. pneumoniae,* we checked whether these new essential genes map to known essential genes in other bacteria. To this end, we used 33 gene essentiality datasets, covering 24 bacterial species, as available in the Database of Essential Genes (DEG) (Luo et al., 2014) (Fig. 2a, right box). We mapped the 52 new essential genes from *K. pneumoniae* (Supplementary Dataset 10; Fig. 2c) to DEG essential genes by using functional annotation and protein sequence comparison (BLASTP). Thirty-eight out of the 52 genes mapped to essential genes in at least 5 experimental datasets with both BLASTP and functional annotation. Similarly, 21 genes mapped, with both of the searching methods, to 11 or more datasets (one third of the total datasets, spanning 8 or more different species) where these genes were experimentally determined as essential (Fig. 2c, Supplementary Dataset 11).

The vast majority of the new essential genes (44) are annotated to functions related with biosynthesis of cofactors and prosthetic groups (Fig. 2c). Moreover, all of the 21 genes mapped to at least one third of the experimental essentiality datasets belong to that metabolic subsystem. For the subset of 44 cofactor-associated new essential genes, the median presence of a gene in DEG datasets is 31.8%; when additionally narrowing the searched DEG datasets for γ-Proteobacteria only (the class of *K. pneumoniae*), the median presence of a gene increases to 50% (Fig. 2d). This indicates that the BOF swap introduced new cofactors on the objective function that are highly likely to be essential. New essential genes matching with half or more the γ-Proteobacteria datasets on DEG relate to isoprenoids (ispD, ispE, ispF, ispG, ispH, ispB), coenzyme A (coaE, coaD, dfp), folates (folE, folB), heme (gltX, hemC, hemE, hemD, hemL, hemB, hemG), flavins (ribF, ribA, ribD) and more than one cofactor (dxs, dxr) biosynthesis. The associated full reactions names in KEGG and amino acid sequences are available in Supplementary Dataset 11.

### 2.4 Integration of multiple data sources reveals universally essential cofactors

**Fig. 3:**
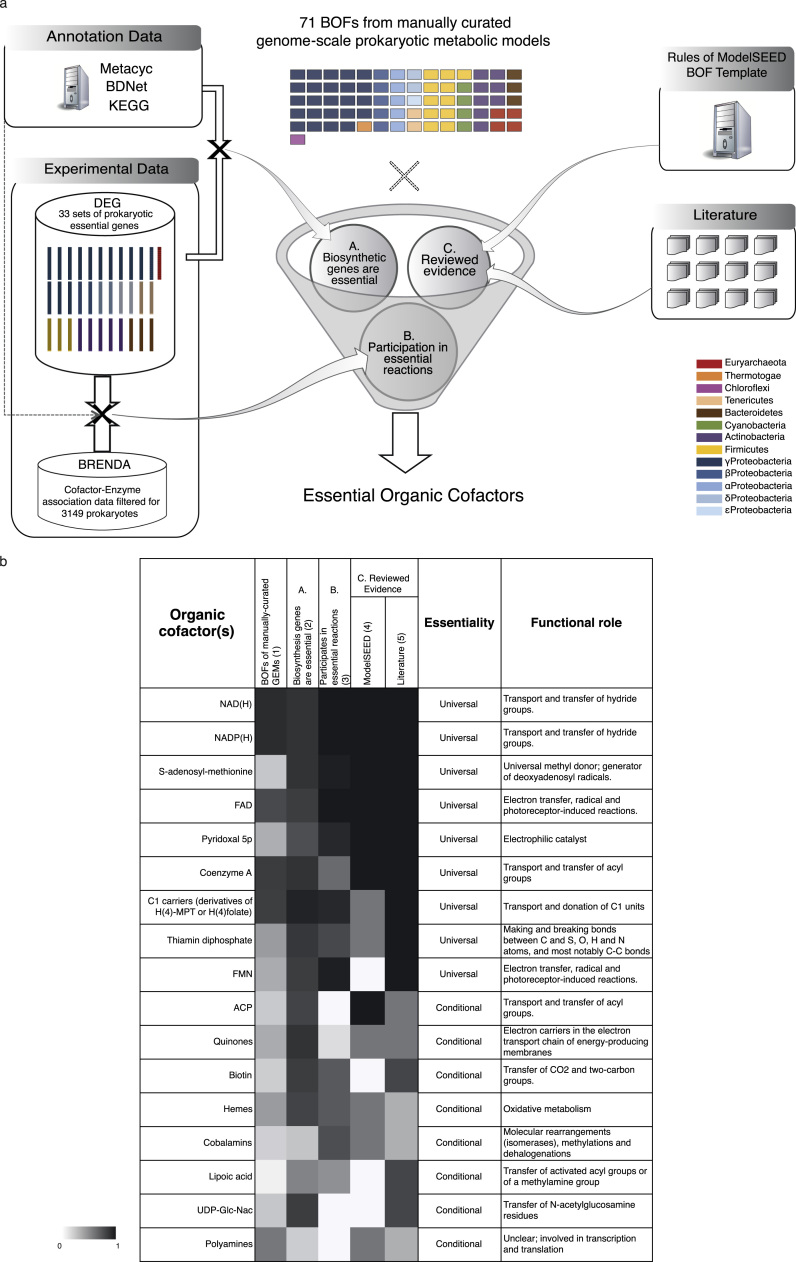
**Essential cofactors for prokaryotic metabolism.** (a). Data integration pipeline used towards identification of universally and conditionally essential cofactors. Color-code of BOF and DEG datasets according to phyla. (b) Prevalence of high-ranking prokaryotic essential cofactors, or classes thereof, in different analyses. Cofactor classes were defined after data integration as sets of functionally related molecules for which at least one representative should be chosen for simulations of biomass production. Capital letters A, B and C refer to the levels of evidence shown in (a). ModelSEED scores: 1 - universal; 0.5 - conditional; 0 - not in the template. Literature scores: 1 - no exception found in the literature; 0.75 - several essentiality cases reported but at least one exception found; 0.25 - several exceptions found. See Supplementary Information for full descriptions of exceptions.

The true-positive (experimentally essential) rate of cofactor-related essential genes of iYL1228 in γ-Proteobacteria when using the biomass composition of iAF1260 indicates organic cofactors as crucial but also missing biomass components in prokaryotic GEMs. To close this gap, we set out to identify universally essential cofactors (or classes thereof) for prokaryotes that will improve accuracy and comparability of GEMs. For this, we integrated multiple large-scale datasets (Fig. 3a). The compositions of cofactor pools of GEMs (Supplementary Dataset 12) were not used as evidence due to the lack of biological consistency and standards mentioned above. We used three levels of evidence. A: the essentiality of genes involved in the biosynthesis of the cofactor(s) (Supplementary Datasets 13 and 14). B: the participation of the cofactor(s) in reactions catalyzed by essential enzymes as per the enzyme-cofactor association data from BRENDA (Chang et al., 2015) (Supplementary Datasets 15–17). C: reviewed evidence, including the ModelSEED template (Supplementary Dataset 5) and an extensive review of publications on prokaryotic organic cofactors (Supplementary Table 1; Supplementary Discussion). Each level of evidence was scored on a scale from 0 to 1. The results, summarized in Fig. 3b, indicate 8 universally essential cofactors – nicotinamide adenine dinucleotide (NAD), nicotinamide adenine dinucleotide phosphate (NADP), S-adenosyl-methionine (SAM), flavin adenine dinucleotide (FAD), pyridoxal 5-phosphate (P5P), coenzyme A (COA), thiamin diphosphate (THMPP) and flavin mononucleotide (FMN) plus one class of cofactors, which we identified as C1 carriers (includes tetrahydrofolates for bacteria and tetrahydromethanopterins for most archaea). Highly essential cofactors with less evidence and for which there are some known exceptions were classified as conditionally essential cofactors, in which case we identified either the phylogenetic branch not requiring this cofactor (e.g. most archaea do not use ACP) or metabolic modes in which it is not essential. In the Supplementary Information we discuss this classification and summarize metadata on functional roles, alternative nomenclature, related compounds, known transport systems and specificities that illustrate the complexity of the cofactor usage in prokaryotes.

### 2.5 New pathways and improved gene essentiality predictions for *Mycobacterium tuberculosis*

**Fig. 4:**
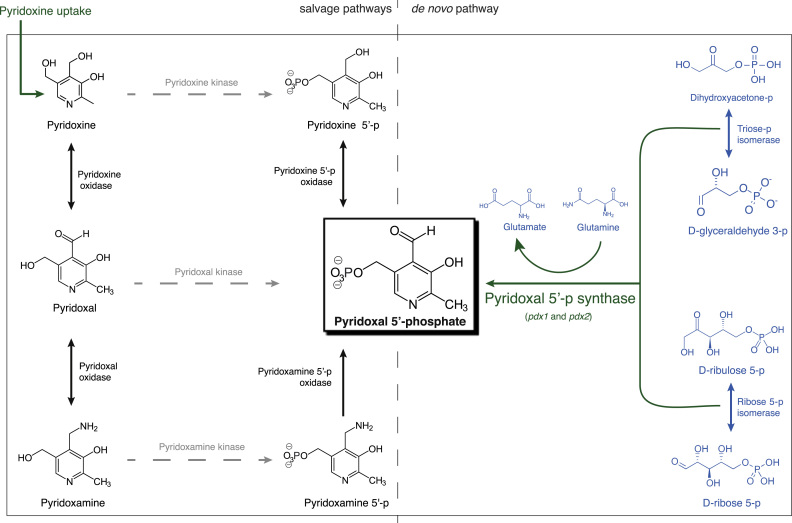
**Pyridoxal 5′-phosphate (P5P) production in*****Mycobacterium tuberculosis***. In black, the compounds and reactions present in genome-scale models iNJ661, iNJ661m and iNJ661v. In blue, reactions and compounds present in these models and also in GSMN-TB. In green, additions of this work to iNJ661v that permit *de novo* production of P5P, which was not possible with any of the existing models. In grey and dashed arrows, reactions for which there is indirect biochemical evidence and no genetic evidence for *M. tuberculosis.*

To substantiate our proposal of essential cofactors for prokaryotic life, we chose the genome-scale model of *Mycobacterium tuberculosis* iNJ661v (Fang et al., 2010), a species for which there exists comprehensive experimental data for validations of predictions (Sassetti et al., 2003). Furthermore, although several GEMs have been built and improved for *M. tuberculosis* (Beste et al., 2007, Fang et al., 2010, Jamshidi and Palsson, 2007), none of the BOFs include all of the here-proposed universally essential cofactors (conditionally essential cofactors were excluded from this analysis). In iNJ661v, the most recent of all, although the BOF was missing NAD, NADP, COA, FAD, FMN, SAM and P5P, the network was able to produce all of these cofactors with the exception of P5P. To resolve the latter, we searched the literature for the known biochemistry regarding P5P in *M. tuberculosis.* Indeed, we found experimental evidence not only for a *de novo* pathway for its production that was missing in the model, but also for the essentiality of P5P for growth, survival and virulence of *M. tuberculosis* (Dick et al., 2010). After completing the BOF with all the mentioned universal cofactors that were missing, we added the new biosynthetic reaction of P5P to the model and the associated two biosynthetic genes. This completed picture of P5P biosynthesis in *M. tuberculosis* is shown in Fig. 4. The experimental study by Dick et al., that validated the P5P *de novo* pathway, reports that the growth of a mutant in this pathway could be rescued when providing pyridoxine in the medium (Dick et al., 2010). This indicates that one or all of the phosphorylations of pyridoxine, pyridoxamine or pyridoxal for which there is no genetic evidence must occur, and the gene(s) encoding them remain to be discovered. To test the modified model for its ability to predict gene essentiality, we simulated single gene knockouts in several media, including an *in silico* medium mimicking Middlebrook media (used in the experimental assay for validation of the predictions (Sassetti et al., 2003); Online Methods). Indeed, the gene essentiality predictions improved for the cofactor metabolic pathways, with 7 new true predictions with the completed model (Supplementary Datasets 18 and 19). The corresponding proteins are also expressed in *M. tuberculosis* (Schubert et al., 2013), adding more evidence to our findings.

## 3 Discussion and conclusions

Answering the question of what to include in the core of a biomass objective function is not always straightforward. One example is different nucleotide forms, which, although inter-convertible, are essential for cellular chemistry. We propose here that all essential and irreplaceable molecules for metabolism should be included in the biomass functions of genome scale metabolic models. In the special case of cofactors, when two forms of the same cofactor take part in the same reactions (such as NAD and NADH), only one form could be included for the sake of simplicity. When a class of cofactors includes active and non-active interconvertible forms, the active forms should be preferred. A simple example case is the representation of flavins: FAD and FMN are the preferred active forms to be included in the BOF, oppositely to riboflavin, the non-active precursor. More details on the different approaches on modeling biomass compositions are discussed in the Supplementary Discussion.

The comparison of gene essentiality in different species has some limitations. These can occur due to biological causes, as the alternative presence of isozymes and transporters that introduce redundancy in different networks, and different media conditions where the large-scale assays were performed. Other limitations can occur due to errors and incompleteness in databases, which we examine further in the Supplementary Discussion. By overlapping several levels of evidence in the prediction of essential cofactors (Fig. 3) our approach is conservative, in order not to introduce false positive (non-essential *in vivo*) predictions.

We here propose a standardized and detailed core biomass composition for prokaryotes (Supplementary Fig. 1). This is a conservative proposal and thus includes only the three most prevalent lipid components as representative species (phosphatidylglycerol, phosphatidylethanolamine and cardiolipin), and these should be adapted according to the species being modeled. Our proposal excludes other non-universal macromolecules such as cell wall peptidoglycans (more details can be found in the Supplementary Discussion). We further suggest that the pipeline used here (Fig. 3a) can be expanded, in the future, in the formulation of new BOFs of organisms for which there are experimental essentiality data for the species or for phylogenetically-close species, as genome-scale experimental essentiality datasets keep being expanded; all three levels of evidence used here can be adapted to scan components other than organic cofactors.

The cofactors here identified as universally essential play fundamental roles in biochemistry. In most cases, they are related with the transfer of small units: hydride groups for NAD(P)(H), methyl groups for SAM, electrons for FAD and FMN, acyl groups for CoA and one-carbon units in C1 carriers. The two special cases of P5P and THMPP correspond to direct intervention in catalysis, stabilizing intermediate metabolites and assisting in the formation of new chemical bonds, respectively. Our classification of universally essential is conservative, excluding cofactors for which we found minor exceptions in the data analyzed, e.g. biotin (Fig. 3b; Supplementary Information). Such exceptions could be false predictions of non-essentiality due to incomplete data or biases in databases, e.g. interactions in BRENDA may exclude carrier cofactors like CoA, ACP and quinones (more details in the Supplementary Discussion).

Updating the biomass composition in metabolic models allowed us to identify new candidate essential genes for *K. pneumoniae* backed by experimental genetic evidence for orthologs of related species. These could serve as potential drug targets for *K. pneumoniae,* a pathogen causing urgent concerns regarding antibiotic resistance (Kontopidou et al., 2014, Snitkin et al., 2012). We also demonstrate the importance of using a comprehensive biomass composition for *M. tuberculosis*. Our modifications successfully led to the identification of a previously validated pathway for vitamin B6 biosynthesis, which was missing in the current models, and improved gene essentiality predictions.

When a new (essential) component is included in the BOF, it implies that this component needs to be provided, either through the biosynthetic pathway or via transport reactions. The construction of more complete and standardized BOFs will thus have a great impact not only in the predictions of essential genes but also in the construction of minimal media required for growth. Both applications are of utmost importance for identifying metabolic vulnerabilities of pathogens, being in fact the most common motivations to construct GEMs for those organisms.

Overall, this work lays foundations for improving the definition of biomass composition in the current and future metabolic reconstructions – an important step towards biochemically more accurate models with higher predictive power. Moreover, it is the first large-scale systematization of essential metabolic organic cofactors for prokaryotes, which we hope will be useful for several fundamental and applied studies.

## 4 Material and methods

### 4.1 Collection and comparison of detailed BOFs in GEMs

We searched for manually-curated GEMs of prokaryotes in four major online databases: BiGG (Schellenberger et al., 2010), MetRxn (Bernard et al., 2014), BioModels (Chelliah et al., 2015) and GSMNDB (Systems Biology and Metabolic Engineering Research Group at the Tianjin University, 2014); and in an updated list of GEMs as per Palsson group website (Systems Biology Research Group at the University of California San Diego, 2014) (accession date: March 2014). The biomass composition was, whenever possible, retrieved directly from the model file; if the model was not available or not accessible, the composition, along with the metadata, was taken from the publication (Supplementary Dataset 1). For the cases where several important macromolecules or the solute pool were represented in lumped reactions, we deconstructed the composition from the individual lumped reactions. For nomenclature standardization, we created an initial list with all the metabolites from BOFs of GEMs built with BiGG nomenclature. Each individual component of all remaining BOFs was matched against that list, with the help of mappings of ModelSEED (Henry et al., 2010). The non-matching metabolites were checked manually for matches with alternative names. Several species-specific tagged metabolites were discarded, although if they could be matched as generalist lipids (e.g. phosphoethanolamine) or peptidoglycan the tag would be removed or the id would be substituted by the more general id. For yet non-matching metabolites, a new entity and id was created in the list (Supplementary Datasets 2–4).

The ModelSEED template for universal biomass components was obtained from the original publication (Henry et al., 2010).

### 4.2 Cluster analysis

Hierarchical clustering was performed using ‘pvclust’ R package (Suzuki and Shimodaira, 2006) with binary distance as a dissimilarity metric and Ward's method as the linkage criterion. For accessing uncertainty, approximately unbiased p-values were calculated via multiscale bootstrap resampling. All statistical analyses were performed using R statistical software version 3.1.

### 4.3 BOF swap

We chose five different GEMs by sampling high and low phylogenetic dissimilarity pairs in order to assess the impact of BOFs in predictions of essentiality (Fig. 2a-b; Supplementary Dataset 1). When adding a new BOF to a model, we verified that the model contained and could produce all the new metabolites added, and if not, those were removed from the BOF (Supplementary Dataset 8). We also checked that the wild-type network was viable with all the existing import reactions set to a positive value (20 mmol/gDW/h). Often some metabolites were not added, either for not being represented in the model at all, or for being end-points of blocked pathways in the network. The same media conditions were used for simulations before and after all swaps. The swaps likely alter the interpretation (units) of biomass in the BOF, which however does not affect the Boolean results of feasibility of biomass production. Essentiality predictions with the new BOFs were classified as new negatives (essential with the original BOF, but not with the new BOF) or new positives (non-essential in the original model but essential with the new BOF) or as same predictions (see Fig. 2a).

### 4.4 Simulations of reaction/gene deletion phenotypes

Simulations of maximum growth rates for single-deletions of reactions and genes were performed using Flux Balance Analysis (FBA) (Savinell and Palsson, 1992, Varma and Palsson, 1993a). All modeling procedures were implemented in C++ and solved using IBM ILOG CPLEX solver.

### 4.5 Mapping *in silico* essential genes with large-scale experimental essential datasets

Searches in DEG (Luo et al., 2014) were performed manually for each of the 52 new essential genes of iYL1228 to find possible true positives – experimentally essential genes that were predicted as essential in the simulation (Fig. 2a). Matching was done by searching for the corresponding gene annotation and, independently, with BLASTP in DEG with an E-value threshold of 10e-6.

### 4.6 Cofactor usage/biosynthesis data

We extracted all enzyme-cofactor association data for prokaryotes using the Python SOAP access methods for BRENDA (Chang et al., 2015). Biosynthetic genes for each cofactor or class of cofactors identified in the cross-integration of DEG and BRENDA were extracted manually from Metacyc (Caspi et al., 2014). For the mapping of gene names in DEG with BRENDA and Metacyc, bioDBNet (Mudunuri et al., 2009) and KEGG (Kanehisa et al., 2014) were used.

### 4.7 Modification of iNJ661v

All changes described in the main text were performed manually on the original SBML file for iNJ661v. To simulate Middlebrook media as used in the genome-scale experimental assay for validation of the predictions (Sassetti et al., 2003), new transporters for biotin and pyridoxine were added. We set the upper bound of all the respective uptakes of the constituents to 20 mmol/gDW/h, with the exception of albumin, zinc, catalase and oleic acid (not modeled).

## Appendix A Supplementary material

Supplementary material: Supplementary Information: Supplementary Discussion and References, Supplementary Table 1 and Supplementary Figure 1..

.

Supplementary material: Supplementary Datasets 1–6: GSM collection and nomenclature mappings.

.

Supplementary material: Supplementary Datasets 7–9: Supplementary Datasets on BOF swaps.

.

Supplementary material: Supplementary Datasets 10–11: Supplementary Datasets on *Klebsiella pneumoniae* new essentials and mapping with DEG.

.

Supplementary material: Supplementary Datasets 12–17: Supplementary Datasets on the Pipeline to Find Essential Cofactors.

.

Supplementary material: Supplementary Datasets 18–19: Essentiality predictions by modified iNJ661v.

.

Supplementary material: iNJ661v_modified : iNJ661v model in SBML format with Vitamin B6 biosynthesis, completed BOF with essential vitamins and biotin and pyridoxine transport.
