# Supplementary material for: Integration of Biomass Formulations of Genome-Scale Metabolic Models with Experimental Data Reveals Universally Essential Cofactors in Prokaryotes
Source: Metab Eng. 2017 Jan;39:200–8. doi: 10.1016/j.ymben.2016.12.002 (PMC5249239; doi:10.1016/j.ymben.2016.12.002)
Supplement: Supplementary file 1 — Supplementary material: Supplementary Information: Supplementary Discussion and References, Supplementary Table 1 and Supplementary Figure 1.. [file mmc1.docx]

**Supplementary Information**

“Integration of Biomass Formulations of Genome-Scale Metabolic Models with Experimental Data Reveals Universally Essential Cofactors in Prokaryotes”

Joana C. Xavier^1,2^, Kiran Raosaheb Patil^2^, Isabel Rocha^1^

^1^ CEB - Centre of Biological Engineering, University of Minho, Braga, Portugal

^2^ Structural and Computational Biology Unit, European Molecular Biology Laboratory, Heidelberg, Germany

Supplementary Table 1: Details on essential prokaryotic cofactors from literature analysis 2

Supplementary Discussion 7

1. Sensitivity to Errors and Incompleteness in Databases 7

2. Universally Essential Cofactors 7

NAD(H) and NADP(H) 7

S-adenosyl-methionine (SAM) 8

FAD and FMN 8

Pyridoxal-5-phosphate (P5P) 8

Coenzyme A (CoA) 9

C1 Carriers 9

Thiamin diphosphate 9

3. Conditionally Essential Cofactors 9

Acyl-carrier protein (ACP) 9

Quinones 10

Biotin 10

Hemes 10

Cobalamins 11

Lipoic Acid 11

UDP-Glc-Nac 11

Polyamines 11

4. Other details on modeling biomass compositions 11

Supplementary Figures 12

Supplementary References 13

# ****Supplementary Table 1:** Details on essential prokaryotic cofactors from literature analysis**

| **Cofactor or Class of Cofactors** | UNIVERSALLY ESSENTIAL | | | | | | | | | | | | | |
| --- | --- | --- | --- | --- | --- | --- | --- | --- | --- | --- | --- | --- | --- | --- |
|  |  |  |  |  |  |  |  |  |  |  |  |  |  |  |
|  | **DI-NUCLEOTIDES (B3)** | | **CoA (B5)** | **FLAVINS (B2)** | | | | **C-1 carriers - derivatives of tetrahydrofolate (B9) or tetrahydromethanopterin** | | | | **Thiamin diphosphate (B1)** | **Pyridoxal phosphate (B6)** | **SAM** |
| **Name and ID** | **Nicotinamide-adenine dinucleotide (nad)** | **Nicotinamide-adenine dinucleotide phosphate (nadp)** | **Coenzyme A (coa)** | **Flavin adenine dinucleotide (fad )** | **Flavin mononucleotide (fmn)** | Riboflavin (ribflv) | f420 | Tetrahydrofolate (thf) | 10-Formyltetrahydrofolate (10fthf) | | 5-Methyltetrahydrofolate (5mthf) | **Thiamin diphosphate (thmpp)** | **Pyridoxal 5'-phosphate (pydx5p)** | **S-adenosyl-methionine (amet)** |
| **Literature supporting universal essentiality** | (1, 2) | (2, 3) | (1, 4) | (1, 5) | (5, 6) |  |  | (1, 7, 8) | | | | (1, 9–11) | (1, 12–14) | (15–17) |
| **ModelSEED** | Universal | Universal | Universal | Universal | NO | Universal | NO | Conditional (not in Archaea) | | NO | NO | Conditional | Universal | Universal |
| **Functional Role** | Transport and transfer of hydride groups. | | Transport and transfer of acyl groups. | Electron transfer, radical and photoreceptor-induced reactions. | Electron transfer, radical and photoreceptor-induced reactions. | Precursor to FAD and FMN that can be uptaken; does not have biological activity (18). | Electron transfer in methanogens, actinobacteria, and others | Transport and donation of C1 units (7, 19) | | Synthesis of the purine ring; formation of the initiator of protein synthesis, fMet-tRNA (19) | Methionine synthesis (sustaisn AdoMet turnover) (19) | Assists in making and breaking bonds between carbon and sulfur, oxygen, hydrogen, and nitrogen; and most notably C-C bonds (9) | Electrophilic catalyst, stabilizes intermediates in a.a. metabolism and is also a cofactor for glycogen phosphorylases (13) | Universal methyl donor, key element in the "methylation cycle". Generator of deoxyadenosyl radicals. |
| **Alternative names** | Nadide; Diphosphopyridine nucleotide; DPN;Nicotinamide adenine dinucleotide;NAD+ | Triphosphopyridine nucleotide;TPN;beta-Nicotinamide adenine dinucleotide phosphate;Nicotinamide adenine dinucleotide phosphate | CoA-SH | Flavinadeninedinucleotideoxidized; Flavin adenine dinucleotide oxidized | riboflavin-5′-phosphate | RIBF;Vitamin B2;7,8-Dimethyl-10-ribitylisoalloxazine;Lactoflavin | 8-hydroxy-5-deazaflavin | 5-6-7-8-Tetrahydrofolate;(6S)-THFA;(6S)-Tetrahydrofolic acid;(6S)-Tetrahydrofolate;THF;Tetrahydrofolic acid;5,6,7,8-Tetrahydrofolate | |  |  | thiamine pyrophosphate ;ThPP;TPP | Pyridoxal 5'-phosphate;Pyridoxal 5-phosphate;Pyridoxal phosphate; Vitamin B6 ;PLP | S-Adenosyl-L-methionine; SAM-e; SAMe, AdoMet, ademethionine |
| **Other representatives of the same class used in GSMs (id)** | NADH; nicotinic acid (Na), nicotinamide (Nam) and nicotinamide riboside (NR);nicotinamide mononucleotide (NMN) | NADPH | accoa, succoa, malcoa; pantothenate | fadh2 | FMNH2 |  |  | 5,10-Methylenetetrahydrofolate - mlthf; 5-Formyltetrahydrofolate - 5fthf | | | | Thiamin; Thiamin triphosphate (ThTP) | Pyridoxine (PN); Pyridoxine 5'-phosphate (PNP); Pyridoxal (PL); Pyridoxamine (PM); Pyridoxamine 5'-phosphate (PMP) |  |

**Supplementary Table 1 *(continued)***

| **Transport system exists** | Yes (20); for niacin (21) ; for other precursors nicotinamide riboside (NR) and nicotinamide mononucleotide (NMN) (22) | Precursor (pantothenate), imported by virtually all organisms (23). CoA uptake has been hypothesized in intracellular parasites lacking biosynthetic ability (24, 25) | For riboflavin (precursor) (5), specifically in *Rhizobium leguminosarum* and other proteobacteria (26) and Spirochetes (27) | | |  | Firmicutes; predicted for some pathogens and cyanobacteria (28, 29); Lactobacillus brevis (30) | | | Thiamin (31) | Salmonella typhimurium LT2 (32) Lactic acid bacteria(33) Ecoli (34) Streptococcus mitior (35) | Several species (17, 36, 37) |
| --- | --- | --- | --- | --- | --- | --- | --- | --- | --- | --- | --- | --- |
| **Essential for other functions than metabolism** | Several signaling events (covalent protein modifications, protein deacetylation, precursors of the intracellular calcium-mobilizing agents and regulation of transcription) (2) |  |  |  |  |  |  |  |  |  | Protective agent against reactive oxygen species (38) | Regulation of gene expression (39); Assembly of the septal ring in cell division (16, 40, 41); Transcription termination control (42) |
| **Interesting facts and studies related to metabolism** | No promiscuity between the two pairs (NADH-NAD+ and NADPH-NADP+) in enzymes (43); "Determining the Extremes of the Cellular NAD(H) Level" (44) | It has been estimated that 4% of all enzymes use CoA or a derivative (45) |  |  |  | F420 may confer an advantage to mycobacteria in anaerobic environments because it has a lower redox potential than NADP (6) | Some archaea use tetrahydromethanopterin (H4-MPT) or dephospho-H4-MPT, but others use folates (7); 5mthf is the dominant folate species (19) ; "Folate levels in cultures of lactic acid bacteria" (46) | | |  | Arguably the most versatile organic cofactor in biology; 1.5% of all genes in prokaryotes code for PLP-dependent enzymes (13); E. coli can bypass a block in pyridoxal‐5′‐phosphate synthesis (47); “Phylogenetic analyses and comparative genomics of vitamin B6 (pyridoxine) and pyridoxal phosphate biosynthesis pathways."(48) | SAM synthases are quite different in Archaea (49) Bacto-eukaryal SAM Synthase has a unique fold (50) SAM-synthase is induced by leucine (40); 80% of the free Met present in the cell is used in the methylation cycle that continuously supplies with the dozens of methyltransferases in the cell with SAM (19) |

**Supplementary Table 1 *(continued)***

| **Cofactor or Class of Cofactors** | CONDITIONALLY ESSENTIAL | | | | | | | | | | | | | |
| --- | --- | --- | --- | --- | --- | --- | --- | --- | --- | --- | --- | --- | --- | --- |
|  |  |  |  |  |  |  |  |  |  |  |  |  |  |  |
|  | **POLYAMINES** | | | **Hemes** | **Terpenoid (or isoprenoid) quinones** | | | | | **Biotin (B7 or H)** | **ACP** | **Cobalamins (B12)** | **Lipoyl Moyeties** | **UDP-GlcNAc** |
| **Name and ID** | Putrescine (ptrc) | | Spermidine (spmd) | Protoheme (pheme) | Menaquinone (mqn_n) | | Ubiquinone (q_n) | | Methionaquinone (mtk) | Biotin (btn) | Acyl-carrier protein (ACP) | Adenosylcobalamin (adocbl) | Lipoic acid | uridine diphosphate N-Acetylglucosamine (UDP-GlcNAc) |
| **ModelSEED** | Conditional | | Conditional | Conditional | Conditional | | Conditional | | NO | NO | Universal | Conditional | NO | NO |
| **Functional Role** | Still not very clear; transcription and translation; membranes of Gram - (51) | | | Oxidative metabolism: O2 transport, electron-transport-dependent oxidative phosphorylation, oxidative stress responses, O2-sensing, oxygenation reactions and detoxification; also involved in the synthesis or sensing of other diatomic gases such as carbon monoxide and nitric oxide (52) | Electron carriers in the electron transport chain of energy-producing membranes | | | | | Transfer of CO2 and two-carbon groups. | Transport and transfer of acyl groups. | Molecular rearrangements (isomerases), methylations and dehalogenations | Transfer of activated acyl groups in 2-oxoacid dehydrogenase complexes or of a methylamine group in the glycine cleavage system (53) | transfer of N-acetylglucosamine residues |
| **Alternative names** | PUTR; Tetramethylenediamine; 1,4-Diaminobutane; 1,4-Butanediamine; diamine | N-(3-Aminopropyl)-1,4-butane-diamine; triamine | | Protoheme IX;Heme B;Protoheme;Haem;Heme | Vitamin K2; derivatives of 2-methyl-1,4-naphtoquinone in which the 3-position carries a polyisoprenyl chain of variable length n (named accordingly as MK-n). If having a partially hydrogenated side chain, named as MK-n(Hx) where x is the nr of saturating hydrogen atoms | Coenzyme Q; derivatives of 2,3-dimethoxy-5-methyl-1,4-benzoquinone with a polyisoprenyl side chain of variable length n at the 6-position (named accordingly as Q-n). If having a partially hydrogenated side chain, named as Qn(Hx) where x is the nr of saturating hydrogen atoms. | | 2-methylthio-1, 4-naphthoquinone; similar to menaquinones, but with sulfur (Ishii et al. 1983) | | Coenzyme R |  | Funacomide;Dibencozide;DMBC coenzyme;Coenzyme B12;Calomide;5,6-Dimethylbenzimidazolyl-Co-5'-deoxy-5'-adenosylcobamide;5'-Deoxy-5'-adenosyl-5,6-dimethylbenzimidazolylcobamide;5'-Deoxy-5'-adenosyl vitamin B12;5'-Deoxy-5'-adenosylcobalamin;alpha-(5,6-Dimethylbenzimidazolyl)cobamide coenzyme;(5,6-Dimethylbenzimidazolyl)cobamide coenzyme;(5'-Deoxy-5'-adenosyl)cobamide coenzyme;5,6-Dimethylbenzimidazolyl-5-deoxyadenosyl-cobamidecoenzyme;Cobamamide;Deoxyadenosylcobalamin;Adenosylcobalamin;Cobamide coenzyme | Lipoyl-protein or lipoyl-domain; lipoamide; Thioctic acid;alpha-Lipoic acid;Lipoate ;Lipoamide | UacGAM; udp-n-acetyl-d-glucosamine; udpnag; |

**Supplementary Table 1 *(continued)***

| **Other representatives of the same class used in GSMs (id)** | spermine; cadaverine | siroheme;hemeO;ferriheme; hemeA; hemeD | dimethylmenaquinone (DMKs) plastoquinones (PQ) caldariellaquinone (CQ). | Biotinyl-5-AMP (btamp) | phosphopantetheine | methylcobalamin; adocobalamin | protein bound lipoate (lipopb) | UDP-N-acetylmuramate ; UDP-N-acetyl-D-galactosamine; |
| --- | --- | --- | --- | --- | --- | --- | --- | --- |
| **Known cases where it is dispensable** | *Escherichia coli* (54, 55); *Yersinia pestis* (56); Several bacteria, for which growth just gets slower but possible (57) | Facultative anaerobes that use nitrate as terminal electron acceptor under aerobic and anaerobic conditions without heme requirements, with cytochrome systems consisting of metalloflavoproteins (e.g., Escherichia coli and Pseudomonas) (58) Lactic acid bacteria (59–61)(; Other opportunists and symbionts e.g. Streptococcus agalactiae, Streptococcus pyogenes, Enterococcus faecalis and Buchnera aphidicola (62); Treponema pallidum, Borrelia burdorferi, or Mycoplasma pneumoniae: bacteria that lack heme biosynthesis enzymes, do not seem to express any heme-containing protein and reportedly lack iron transport systems and most common iron proteins (62); Mutants: E.coli (63). Staphylococcus aureus (64). | Methanogens (65–67) | *Buchnera sp*., *Borrelia burgdorferi*, *Aeropyrum pernix*, thermoplasmas, and mycoplasmas have neither the biotin biosynthetic genes nor birA, a bifunctional protein which acts both as a biotin–protein ligase and as a transcriptional repressor of the biotin operor (68). An early book-review reported that some strains of Mycoplasma have a biotin requirement, and others do not (69). | Most archaea (70) | Most obligate pathogenic bacteria and Aquifex aeolicus (71);  Under standard laboratory conditions, E. coli and related species of bacteria (72) | *Helicobacter pylori* (73); Anaerobic Archaea (74) | Mycoplasma (75, 76). |
| **Transport system exists** | Several bacteria and Archaea (51, 77) | Several (62, 78) |  | Several (79, 80) |  | Widely distributed in bacteria and archaea (71) | Yes, and also for precursor (octanoate) (73, 81) |  |

**Supplementary Table 1 *(continued)***

| **Particular cases where essentiality was demonstrated** | *Haemophilus influenzae* (82) |  |  | *E. coli* (83) |  | In almost every enteric bacterium, with the exception of *Escherichia coli*, it is essential for the anaerobic fermentation of 1,2-propanediol, ethanolamine and glycerol (84) ; Methyl transfer in methane-producing archaea (84) *E. coli,* during aerobic growth, when ethanolamine is the sole nitrogen source (72);All prokaryotes that express only class II ribonucleotide reductases (85); Rhodocyclus purpureus (86). |  |  |
| --- | --- | --- | --- | --- | --- | --- | --- | --- |
| **Essential for other functions than metabolism** | Biofilm production in Yersinia pestis (56) and Vibrio cholerae (87) | Regulation (52) | Biosynthesis of pyrimidine compounds in *E.coli;* coupled ATP synthesis, glycosylation of certain membrane proteins early events in sporulation of *B. subtilis* (88) |  |  | Regulation through riboswitches (89) |  |  |
| **Interesting facts and studies related to metabolism** | Polyamines constitute the major polycations in cells together with Mg2+ (90); the distribution is so specific that allows chemotaxonomy (91).  “Distribution of polyamines in methanogenic bacteria." (92) | "A novel pathway for the biosynthesis of heme in Archaea: genome-based bioinformatic predictions and experimental evidence” (93) | Very specific distribution, allowing chemotaxonomy. (94, 95) The quinone content of a bacterial strain changes depending on the growth phase and culture conditions and can be highly correlated with microbial biomass (96).  "An Alternative Menaquinone Biosynthetic Pathway Operating in Microorganisms" (97) "The Futalosine Pathway Played an Important Role in Menaquinone Biosynthesis during Early Prokaryote Evolution" (98) | "Biotin in microbes, the genes involved in its biosynthesis, its biochemical role and perspectives for biotechnological production" (99) | Some pathogens are able to import lipids from their hosts, but they still need ACP to do acylation (100); A few unrelated archaeal species have ACP and the ACP-processing machinery; phylogenetic analysis suggests that they acquired them by HGTs from bacteria (70) | The full chemical synthesis of vitamin B12 is highly complicated, with about 70 steps, and therefore today vitamin B12 is exclusively produced by biosynthetic fermentation processes, using selected and genetically optimized microorganisms (84). | Lipoate is assembled in the cell covalently bound to the enzyme complexes; few lipoate is free in the cytoplasm (53) | "Acetamido Sugar Biosynthesis in the Euryarchaea" (101)  "Characterization of a UDP-N-acetylglucosamine biosynthetic pathway encoded by the giant DNA virus Mimivirus." (102) |

# ****Supplementary Discussion****

## 1. Sensitivity to Errors and Incompleteness in Databases

The deduction of essentiality of cofactors in this study is directly related to the results of several genome-scale assays of gene-essentiality stored in the database of essential genes (DEG) (103). On a first level, although most of the experiments are performed under rich media conditions, which benefits the conservative deduction of universal essentiality done here, the heterogeneity of experimental conditions of the assays should be noted, with one dataset of *Salmonella enterica* with conditional essential genes only (determined under different selective conditions of temperature and nutrients)(104). Secondly, not all the datasets in DEG are exhaustive genome-scale assays of essentiality, for example with one dataset of *Pseudomonas aeruginosa* PAO1 consisting of antibiotic resistance genes (105).

DEG datasets indicate non-essentiality for some cofactors that we classify as universally essential. We note some specific cases below. Generally, even without the above-mentioned limitations, error-free datasets would not be guaranteed (as reviewed in (1) and (106)). Transposon mutant libraries, used in the majority of datasets in DEG (103), can overestimate essentiality through the misclassification of very slow growth mutants as lethal phenotypes. Reversely, essential genes might be classified as dispensable if they tolerate transposon insertions being nevertheless transcribed and translated to functional proteins. Even if consisting only of true classifications, single gene knockout mutant libraries are usually not sufficient for the deduction of functional essentiality. This occurs due to the well-known redundancy that is hard-wired in bacterial metabolism, where alternative pathways allow many times for the biosynthesis, salvage and import of several important molecules, including organic cofactors. These pathways are not always known, especially in the case of less studied species. Multiple simultaneous gene-knockouts would be required to confirm some cases of essentiality classification. Nevertheless, the approach here is conservative as it integrates gene essentiality data with different other types of data, deducing as universally essential only the cofactors with a high confidence level from the integrated data.

Regarding BRENDA, only full EC numbers were mapped to essential genes. Also, some cofactors considered here (e.g. ACP and polyamines) are not present in the cofactor-enzyme association datasets in the database. This decreases the level of evidence for some cofactors, and therefore the confidence of the deduction here would probably increase with the addition of this information.

## 2. Universally Essential Cofactors

Here we discuss some details on the classification of universal essentiality for the different cofactors based on the integration of databases described in the main text. For further details, refer to Supplementary Datasets 18 and 19.

### NAD(H) and NADP(H)

The common redox cofactors are accepted as universally essential without controversy; there are no reports in the literature of the dispensability of these cofactors. Several transporters exist for precursors. The dataset of essentiality of *Pseudomonas aeruginosa* PAO1 in DEG, mentioned already as a set of resistance-related genes, is the only dataset integrated with BRENDA in the current work where no essential gene dependent on NAD(H) was found.

### S-adenosyl-methionine (SAM)

As a universal methyl donor and a key element in the "methylation cycle", SAM plays a fundamental role in metabolism. It is also a generator of deoxyadenosyl radicals, a regulator of transcription (42) and a direct intervenient in the assembly of the septal ring in cytokinesis. Integration of DEG and BRENDA always reveals essential genes depending on this cofactor, with the exception of one dataset of *Salmonella*, although others for the same species disagree. The integration of biosynthetic annotation data with essentiality provides moderate evidence for essentiality, probably due to the known existence of several transporters for this vitamin which surpass the necessity of a biosynthetic route in some species or environments (17, 36, 37).

A study with *Escherichia coli* claims depletion of SAM to very low levels using a SAM hydrolase (107). Others reported temperature-sensitive mutants of metK (a gene involved in SAM biosynthesis) that were genetically unstable and required methionine for growth (108). A later study reviewed metK mutants as leaky, resulting in phenotypes as diverse as overproduction of methionine, methionine auxotrophy or complete inability to grow on defined media; however, all of these phenotypes included a residual SAM synthetase activity (40).

Contradicting these results, metK is classified as essential by at least 23 prokaryotic datasets of genome-scale essentiality in rich media, in DEG. Moreover, El-Hajj and colleagues reported in 2013 that the isolation of mutants totally deficient in SAM synthase became possible only with the isolation and cloning of a SAM transporter from *Rickettsia prowazekii* (36) into an *E. coli* plasmid, allowing the metK mutant to grow in rich medium with an exogenous SAM supply (109). El-Hajj and colleagues used this transporter to study SAM metabolism in further detail (16).

### FAD and FMN

Flavins are accepted as the universal currency for electron transfer, radical and photoreceptor-induced reactions. Riboflavin is commonly represented in the biomass objective functions (BOFs) of genome-scale metabolic models (GEMs) even though it is biologically inactive; there are known transporters for this precursor (18, 26).­­ All datasets in DEG show at least one essential enzyme depending on FAD (Supplementary Dataset 16).

### Pyridoxal-5-phosphate (P5P)

Several reviews indicate P5P as universal and essential (12–14), even though only 25% of GEMs include it in the BOF. It binds covalently to its substrates, which can hinder measurements of the free vitamin available in the cell. There are known alternative pathways to the production of this vitamin, which also hinder inference of essentiality from single gene knockout studies (47).

Further experimentation would be required with *Campylobacter jejunii,* as the dataset from DEG used here (110) indicates non-essentiality when crossed with BRENDA, but another study indicates possible essentiality of pdxA, involved in P5P biosynthesis (111). A recent study reported non-essentiality of pdxA and a full depletion of P5P production, achieved with that single deletion (112), although several questions can be posed regarding the use of those results for the purpose of our work: the use of a rich, undefined medium which most probably contains the vitamin or other vitamers or the more than two fold increase in the direct precursor of P5P in an alternative biosynthetic pathway (pyridoxamine 5 phosphate). Moreover, the residual amounts reported in the mutants could be sufficient for growth, as an amount of 6.0 ng/mL of pyridoxal has shown to be the growth-limiting concentration for mutants of *E. coli* (113).

### Coenzyme A (CoA)

As the universal carrier of acyl groups in cells, reported as used by 4% of all known enzymes, CoA is commonly accepted as universally essential (45). Most species in the datasets used here have essentials genes involved in the biosynthesis of this cofactor, with the exception of the species of the genus *Mycoplasma*. It has been postulated that, along with other pathogens as *Rickettsia* and *Chlamydia*, these species can uptake dephospho-CoA (23).

### C1 Carriers

Tetrahydrofolates in bacteria and some Archaea, and tetrahydromethanopterins in some other Archaea play the essential role of transport and donation of one-carbon units in metabolism (7). Our data supports the essential biosynthesis of the active forms of these cofactors for all species analysed in at least one dataset. Regarding essential enzymes in BRENDA depending on these cofactors, all datasets in DEG show at least one, with the exception of the incomplete datasets referred in section one of this note and the dataset for the archaea, which depends on methanopterins not included in BRENDA.

### Thiamin diphosphate

Thiamin diphosphate assists in making and breaking bonds between several atoms in metabolic reactions, most notably C-C bonds (9). As there are transporter systems identified and assayed, especially for Salmonella (reviewed in (31)) the essentiality of biosynthetic genes alone is inconclusive. However, in three cases where the integration of DEG with BRENDA does not reveal essentiality of thiamin, the annotation of essential biosynthetic genes gives evidence of it: *Burkholderia, Campylobacter* and *Mycoplasma*. In the case of *S. aureus*, the requirement of this cofactor has been shown experimentally (114, 115), as for *H. pylori* (116) and *Streptococcus sanguinis* (117).

## 3. Conditionally Essential Cofactors

Cofactors in this section showed a lower average level of evidence for universal essentiality (see Fig. 3b in main text and Supplementary Datasets 14 and 17). Here we discuss the role of these cofactors in prokaryotic metabolism that can justify these results, pointing the cases where they are known to be not essential. Some highly essential organic compounds that do not fit in the traditional classification of organic cofactor are included here following the classification and inclusion adapted to date in different GEMs.

### Acyl-carrier protein (ACP)

ACP shares with CoA the 4-phosphopantetheine moiety, performing the same function as the latter cofactor as a carrier of acyl groups. It does not have the characteristics of most other cofactors, which can justify its absence from BRENDA cofactor association data, but it is considered a cofactor protein, essential for the synthesis of new membrane in all bacteria. It is currently believed that Archaea carry out fatty acid synthesis in an ancient ACP-independent manner, and most species lack ACP and its related enzymes (70). The other species for which there was no evidence from essential biosynthetic genes related with ACP were *Burkholderia pseudomallei*, for which there is experimental evidence of essentiality (118), and for *Bacteroides thetaiotaomicron*. However, in a close relative to the later, *Bacteroides fragilis*, the gene putatively encoding for ACP is essential and thus further experimentation is required regarding that organism.

### Quinones

Quinones are essential for all chemiosmotic (respiratory or photosynthetic) energy-converting systems, allowing for electron movement across membranes, with the exception of those of methanogenic organisms (67). Strict fermentative metabolism does not require quinones, but even though some bacteria that are obligatorily fermentative have lost their ability to synthesize quinones (being the best studied *Lactobacillus*, *Streptococcus* and *Bifidobacterium* (119)), some of the species still retain the biosynthetic pathway (120) and the electron transport chain and respiratory metabolism can be induced by the presence of both environmental quinones and heme (121, 122). Interest has been raising by the fact that cultures of other anaerobes do produce several menaquinones, in sufficient amounts to provide dietary requirements (e.g. *Lactococcus lactis* and *Brevibacteirum* (119)). Given that quinones play other functions in prokaryotic cells, they can actually be essential in some types of fermentative metabolism (123). The phylogenetic distribution of quinones is widespread across prokaryotes (94) and our data indicates essentiality of biosynthetic genes for many of the species in DEG, even those with a versatile metabolism. More studies are required to analyse the essentiality of quinones in versatile conditions.

### Biotin

Biotin plays a crucial role in the transfer of CO_2_ and two-carbon groups, although our data fails to provide evidence of essentiality of this cofactor in several species. It has been reported that *Buchnera sp*., *Borrelia burgdorferi*, *Aeropyrum pernix*, thermoplasmas and mycoplasmas have neither the biotin biosynthetic genes nor birA, a bifunctional protein which acts both as a biotin–protein ligase and as a transcriptional repressor of the biotin operon (68). For Mycoplasma, it was also reported earlier that some strains have a biotin requirement, and others do not (69). In *E. coli* and other species, it was shown that there is a strict requirement for this cofactor (83).

### Hemes

Heme situation in metabolism is very similar to that of quinones, in that it is essential for both the aerobic and anaerobic respiration, and it has also been shown that its biosynthesis is coupled to it (124). In the absence of exogenous heme or the ability to produce it, some species can live on fermentative metabolism. These include lactic acid bacteria and some opportunistic and endosymbiotic species (59, 62). Other species however have been shown to have a strict requirement for heme, including *Porphyromonas gingivalis, Bacteroides fragilis* and *Haemophilus influenzae* either for the activation of cytochrome oxidases, fumarate reductases and catalases; other functions have been identified that might explain the essentiality of heme in some species, as reviewed in (62). For *Escherichia coli*, it has been shown that even though mutants not able to produce heme can grow anaerobically, they cannot do so in the presence of oxygen, as the expression of fermentative enzymes is limited to anaerobic growth conditions by the activity of redox response regulators (63).

### Cobalamins

Adenosylcobalamin, methylcobalamin and adocobalamin are important in isomerization reactions, methylations and dehalogenations. For *E. coli* and related species, they are only strictly essential in specific environments where glycerol, propanediol and/or ethanolamine are important sources of carbon or nitrogen and energy (72). Other cases where essentiality was demonstrated are methanogenic archaea (84) and *Rhodocyclus purpureus*(86). A comparative genomics study identified the absence of cobalamin biosynthetic genes and regulatory elements in most obligate pathogenic bacteria and in *Aquifex aeolicus* (71)*.*

### Lipoic Acid

Also called lipoate, it is essential for several key enzyme complexes in oxidative and one carbon metabolism, including pyruvate dehydrogenase and α-ketoglutarate dehydrogenase (73). It is therefore not strictly essential in facultative anaerobes, anaerobic organisms and the special case of the microaerophilic *H. pylori* (73).

### UDP-Glc-Nac

UDP-n-acetyl-d-glucosamine is not traditionally considered an organic cofactor, but it usually added in GEMs in this category. It is a universal donor in the transfer of N-acetylglucosamine residues, essential for the synthesis of the cell wall in prokaryotes (101). Currently it is accepted that only Mycoplasma does not require this compound, as it does not produce cell wall (76).

### Polyamines

The role of polyamines in prokaryotic metabolism (the most common in Bacteria and Archaea being putrescine and spermidine) is ubiquitous, as reviewed in (51). Our analysis here included them as organic cofactors mainly due to their classification in GEMs, which have included them broadly (49%). However, polyamines act more as stabilizers and signaling molecules and are not usually considered as cofactors (125) (for this reason, as with ACP, no essential enzymes are matched as depending on them). It has been reported that polyamines are essential for normal growth (125), even though their essentiality is not prevalent, with reported dispensability in *E. coli* (54), *Yersinia pestis* (56) and several others (57).

## 4. Other details on modeling biomass compositions

There are few exceptions in which the introduction of an essential component in the BOF might bring additional questions unaddressed in the context of GEMs. This is the case of the acyl carrier protein (ACP) and potentially other protein-based components. ACP is included in the ModelSEED universal template for biomass composition and can be seen as essential in most organisms. However, its inclusion in the BOF implies the introduction of a biosynthetic pathway in the model, which might lead to inconsistencies, as no other protein has a dedicated pathway in GEMs. An alternative would be to include an artificial transport reaction, which implies adding information to the model that has no correspondence in reality.

Another situation with even higher biological relevance is that of RNA. Some models already include tRNA and the essential reactions and genes that charge each individual tRNA molecule with its respective amino acid. There is, however, a generalized absence of mature rRNA, which pool also needs to be maintained stable, by duplicating with each cell division, involving metabolic transformations (126).

The biomass composition of a cell can change with different growth conditions within the same strain (127–130). Setting a standardized average core biomass composition (Supplementary Fig. 1) is only the starting point for increasingly better, more predictive genome-scale metabolic models.

# ****Supplementary Figures****


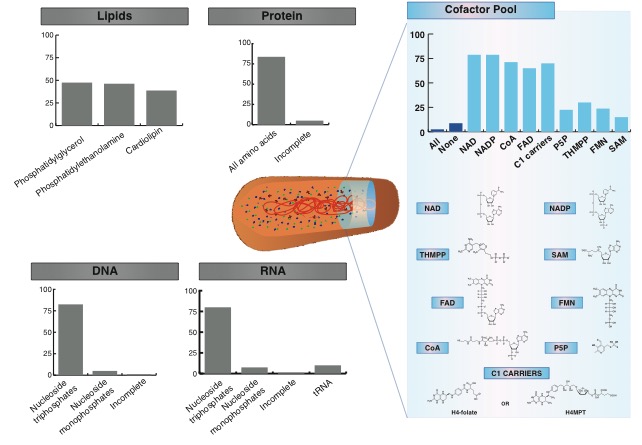


Supplementary Fig. 1 - Most prevalent components in the biomass composition of manually curated genome-scale metabolic models of prokaryotes and a proposal of universally essential organic cofactors. Prevalence in bar plots is quantified as the percentage of the 71 models analyzed in which each specified component or set of components is present. Highlighted set of organic cofactors summarizes the findings of this work: light blue, universal organic cofactors; dark blue, all – number of models containing all cofactors in the BOF; none – number of BOFs containing none of the indicated cofactors.

# Supplementary References

1. Gil R, Silva FJ, Peretó J, Moya A (2004) Determination of the Core of a Minimal Bacterial Gene Set. *Microbiol Mol Biol Rev* 68(3):518–537.

2. Berger F, Ramírez-Hernández MH, Ziegler M (2004) The new life of a centenarian: Signalling functions of NAD(P). *Trends Biochem Sci* 29(3):111–118.

3. Pollak N, Dölle C, Ziegler M (2007) The power to reduce: pyridine nucleotides--small molecules with a multitude of functions. *Biochem J* 402:205–218.

4. Rothmann M, et al. (2013) Metabolic perturbation of an essential pathway: Evaluation of a glycine precursor of coenzyme A. *J Am Chem Soc* 135(Figure 1):5962–5965.

5. Abbas C a, Sibirny A a (2011) Genetic control of biosynthesis and transport of riboflavin and flavin nucleotides and construction of robust biotechnological producers. *Microbiol Mol Biol Rev* 75:321–360.

6. Selengut JD, Haft DH (2010) Unexpected abundance of coenzyme F420-dependent enzymes in Mycobacterium tuberculosis and other actinobacteria. *J Bacteriol* 192(21):5788–5798.

7. De Crécy-Lagard V, et al. (2012) Comparative genomics guided discovery of two missing archaeal enzyme families involved in the biosynthesis of the pterin moiety of tetrahydromethanopterin and tetrahydrofolate. *ACS Chem Biol* 7(11):1807–1816.

8. Levin I, Giladi M, Altman-Price N, Ortenberg R, Mevarech M (2004) An alternative pathway for reduced folate biosynthesis in bacteria and halophilic archaea. *Mol Microbiol* 54:1307–1318.

9. Frank R a W, Leeper FJ, Luisi BF (2007) Structure, mechanism and catalytic duality of thiamine-dependent enzymes. *Cell Mol Life Sci* 64:892–905.

10. Frank R a W, Kay CWM, Hirst J, Luisi BF (2008) Off-pathway, oxygen-dependent thiamine radical in the krebs cycle. *J Am Chem Soc* 130(2):1662–1668.

11. Makarchikov AF, et al. (2003) Thiamine triphosphate and thiamine triphosphatase activities: from bacteria to mammals. *Cell Mol Life Sci* 60(7):1477–88.

12. Fitzpatrick TB, et al. (2007) Two independent routes of de novo vitamin B6 biosynthesis: not that different after all. *Biochem J* 407:1–13.

13. Percudani R, Peracchi A (2003) A genomic overview of pyridoxal-phosphate-dependent enzymes. *EMBO Rep* 4(9):850–854.

14. Christen P, Mehta PK (2001) From cofactor to enzymes. The molecular evolution of pyridoxal-5’-phosphate-dependent enzymes. *Chem Rec* 1:436–447.

15. Epshtein V, Mironov AS, Nudler E (2003) The riboswitch-mediated control of sulfur metabolism in bacteria. *Proc Natl Acad Sci U S A* 100(9):5052–5056.

16. El-Hajj ZW, Reyes-Lamothe R, Newman EB (2013) Cell division, one-carbon metabolism and methionine synthesis in a metK-deficient Escherichia coli mutant, and a role for MmuM. *Microbiology* 159(Pt_10):2036–2048.

17. Haferkamp I, et al. (2013) The endosymbiont amoebophilus asiaticus encodes an s-adenosylmethionine carrier that compensates for its missing methylation cycle. *J Bacteriol* 195:3183–3192.

18. Vogl C, et al. (2007) Characterization of riboflavin (vitamin B2) transport proteins from Bacillus subtilis and Corynebacterium glutamicum. *J Bacteriol* 189(20):7367–7375.

19. Rébeillé F, et al. (2007) Roles of vitamins B5, B8, B9, B12 and molybdenum cofactor at cellular and organismal levels. *Nat Prod Rep* 24:949–962.

20. Reidl J, et al. (2002) NADP and NAD utilization in Haemophilus influenzae. *Mol Microbiol* 35(6):1573–1581.

21. ter Beek J, Duurkens RH, Erkens GB, Slotboom DJ (2011) Quaternary structure and functional unit of energy coupling factor (ECF)-type transporters. *J Biol Chem* 286(7):5471–5.

22. Sauer E, Merdanovic M, Mortimer AP, Bringmann G, Reidl J (2004) PnuC and the utilization of the nicotinamide riboside analog 3-aminopyridine in Haemophilus influenzae. *Antimicrob Agents Chemother* 48:4532–4541.

23. Spry C, Kirk K, Saliba KJ (2008) Coenzyme A biosynthesis: an antimicrobial drug target. *FEMS Microbiol Rev* 32(1):56–106.

24. Genschel U (2004) Coenzyme A biosynthesis: Reconstruction of the pathway in archaea and an evolutionary scenario based on comparative genomics. *Mol Biol Evol* 21(7):1242–1251.

25. Degnan PH, Lazarus AB, Wernegreen JJ (2005) Genome sequence of Blochmannia pennsylvanicus indicates parallel evolutionary trends among bacterial mutualists of insects. *Genome Res* 15:1023–1033.

26. García Angulo V a., et al. (2013) Identification and characterization of ribN, a novel family of riboflavin transporters from rhizobium leguminosarum and other proteobacteria. *J Bacteriol* 195:4611–4619.

27. Deka RK, Brautigam C a., Biddy B a., Liu WZ, Norgard M V. (2013) Evidence for an ABC-type riboflavin transporter system in pathogenic spirochetes. *MBio* 4. doi:10.1128/mBio.00615-12.

28. Eudes A, et al. (2008) Identification of genes encoding the folate- and thiamine-binding membrane proteins in firmicutes. *J Bacteriol* 190:7591–7594.

29. de Crécy-Lagard V, El Yacoubi B, de la Garza RD, Noiriel A, Hanson AD (2007) Comparative genomics of bacterial and plant folate synthesis and salvage: predictions and validations. *BMC Genomics* 8(Figure 2):245.

30. Xu K, et al. (2013) Crystal structure of a folate energy-coupling factor transporter from Lactobacillus brevis. *Nature* 497(7448):268–71.

31. Begley TP, et al. (1999) Thiamin biosynthesis in prokaryotes. *Arch Microbiol* 171:293–300.

32. Mulligan JH, Snell EE (1976) Transport and metabolism of vitamin B6 in Salmonella typhimurium LT2. *J Biol Chem* 251:1052–1056.

33. Mulligan JH, Snell EE (1977) Transport and metabolism of vitamin B6 in lactic acid bacteria. *J Biol Chem* 252:835–839.

34. Hockney RC, Scott TA (1979) The Isolation and Characterization of Three Types of Vitamin B6 Auxotrophs of Escherichia coli K12. *J Gen Microbiol* 110(2):275–283.

35. Schiller NL, Roberts RB (1982) Vitamin B6 requirements of nutritionally variant Streptococcus mitior. *J Clin Microbiol* 15(4):740–743.

36. Tucker AM, Winkler HH, Driskell LO, Wood DO (2003) S -Adenosylmethionine Transport in Rickettsia prowazekii. *J Bacteriol* 185(10):3031–3035.

37. Binet R, Fernandez RE, Fisher DJ, Maurelli AT (2011) Identification and characterization of the Chlamydia trachomatis L2 S-adenosylmethionine transporter. *MBio* 2(3):e00051-11.

38. Mooney S, Leuendorf JE, Hendrickson C, Hellmann H (2009) Vitamin B6: A long known compound of surprising complexity. *Molecules* 14:329–351.

39. Winkler WC, Nahvi A, Sudarsan N, Barrick JE, Breaker RR (2003) An mRNA structure that controls gene expression by binding S-adenosylmethionine. *Nat Struct Biol* 10(9):701–707.

40. Newman EB, et al. (1998) Lack of S-adenosylmethionine results in a cell division defect in Escherichia coli. *J Bacteriol* 180(14):3614–3619.

41. Wang S, Arends SJR, Weiss DS, Newman EB (2005) A deficiency in S-adenosylmethionine synthetase interrupts assembly of the septal ring in Escherichia coli K-12. *Mol Microbiol* 58:791–799.

42. McDaniel BAM, Grundy FJ, Artsimovitch I, Henkin TM (2003) Transcription termination control of the S box system: direct measurement of S-adenosylmethionine by the leader RNA. *Proc Natl Acad Sci U S A* 100(6):3083–8.

43. Hult K, Berglund P (2007) Enzyme promiscuity: mechanism and applications. *Trends Biotechnol* 25(5):231–8.

44. Zhou Y, et al. (2011) Determining the extremes of the cellular NAD(H) level by using an Escherichia coli NAD(+)-auxotrophic mutant. *Appl Environ Microbiol* 77(17):6133–40.

45. Begley TP, Kinsland C, Strauss E (2001) The biosynthesis of coenzyme A in bacteria. *Vitam Horm* 61:157–171.

46. Lin M., Young C. (2000) Folate levels in cultures of lactic acid bacteria. *Int Dairy J* 10(5–6):409–413.

47. Kim J, Kershner JP, Novikov Y, Shoemaker RK, Copley SD (2010) Three serendipitous pathways in E. coli can bypass a block in pyridoxal-5’-phosphate synthesis. *Mol Syst Biol* 6(1):436.

48. Mittenhuber G (2001) Phylogenetic analyses and comparative genomics of vitamin B6 (pyridoxine) and pyridoxal phosphate biosynthesis pathways. *J Mol Microbiol Biotechnol* 3(1):1–20.

49. Graham DE, Bock CL, Schalk-Hihi C, Lu ZJ, Markham GD (2000) Identification of a Highly Diverged Class ofS-Adenosylmethionine Synthetases in the Archaea. *J Biol Chem* 275(6):4055–4059.

50. Kozbial PZ, Mushegian AR (2005) Natural history of S-adenosylmethionine-binding proteins. *BMC Struct Biol* 5(1):19.

51. Schneider J, Wendisch VF (2011) Biotechnological production of polyamines by bacteria: recent achievements and future perspectives. *Appl Microbiol Biotechnol* 91(1):17–30.

52. Panek H, O’Brian MR (2002) A whole genome view of prokaryotic haem biosynthesis. *Microbiology* 148(8):2273–2282.

53. Zhao X, Miller JR, Jiang Y, Marletta MA, Cronan JE (2003) Assembly of the Covalent Linkage between Lipoic Acid and Its Cognate Enzymes. *Chem Biol* 10(12):1293–1302.

54. Hafner EW, Tabor CW, Tabor H (1979) Mutants of Escherichia coli that do not contain 1,4-diaminobutane (putrescine) or spermidine. *J Biol Chem* 254(24):12419–12426.

55. Chattopadhyay MK, Tabor CW, Tabor H (2009) Polyamines are not required for aerobic growth of Escherichia coli: preparation of a strain with deletions in all of the genes for polyamine biosynthesis. *J Bacteriol* 191(17):5549–52.

56. Patel CN, et al. (2006) Polyamines are essential for the formation of plague biofilm. *J Bacteriol* 188:2355–2363.

57. Bitoni AJ, Mccann PP (1987) Inhibition of Polyamine Biosynthesis in Microorganisms. *Inhibition of Polyamine Metabolism: Biological Significance and Basis for New Therapies*, eds McCann PP, Pegg AE, Sjoerdsma A (Academic Press, Inc., London), pp 259–275. 1st Ed.

58. Doelle HW (1975) Anaerobic Respiration. *Bacterial Metabolism*, ed Doelle HW (Academic Press, Inc., London), pp 157–207. 2nd Ed.

59. Lechardeur D, et al. (2011) Using heme as an energy boost for lactic acid bacteria. *Curr Opin Biotechnol* 22(2):143–9.

60. Koreny L, et al. (2012) Aerobic kinetoplastid flagellate Phytomonas does not require heme for viability. *Proc Natl Acad Sci* 109(10):3808–3813.

61. Baureder M, Hederstedt L (2013) Heme proteins in lactic acid bacteria. *Adv Microb Physiol* 62:1–43.

62. Gruss A, Borezée-Durant E, Lechardeur D (2012) Environmental heme utilization by heme-auxotrophic bacteria. *Adv Microb Physiol* 61:69–124.

63. Rompf A, Schmid R, Jahn D (1998) Changes in protein synthesis as a consequence of heme depletion in Escherichia coli. *Curr Microbiol* 37:226–230.

64. Kohler C, et al. (2003) Physiological Characterization of a Heme-Deficient Mutant of Staphylococcus aureus by a Proteomic Approach. *J Bacteriol* 185(23):6928–6937.

65. Thauer RK, Jungermann K, Decker K (1977) Energy conservation in chemotrophic anaerobic bacteria. *Bacteriol Rev* 41(1):100–80.

66. Bond DR, Lovley DR (2002) Reduction of Fe(III) oxide by methanogens in the presence and absence of extracellular quinones. *Environ Microbiol* 4(2):115–24.

67. Schoepp-Cothenet B, et al. (2009) Menaquinone as pool quinone in a purple bacterium. *Proc Natl Acad Sci U S A* 106(21):8549–8554.

68. Rodionov DA, Mironov AA, Gelfand MS (2002) Conservation of the biotin regulon and the BirA regulatory signal in Eubacteria and Archaea. *Genome Res* 12(10):1507–16.

69. Smith PF (1991) 3 – Dynamics of Reproduction and Growth. *The Biology of Mycoplasma*, ed Smith PF (Academic Press, Inc., London), pp 99–161. 1st Ed.

70. Lombard J, López-García P, Moreira D (2012) Phylogenomic investigation of phospholipid synthesis in archaea. *Archaea* 2012. doi:10.1155/2012/630910.

71. Rodionov DA, Vitreschak AG, Mironov AA, Gelfand MS (2003) Comparative Genomics of the Vitamin B12 Metabolism and Regulation in Prokaryotes. *J Biol Chem* 278:41148–41159.

72. Fowler CC, Brown ED, Li Y (2010) Using a riboswitch sensor to examine coenzyme B(12) metabolism and transport in E. coli. *Chem Biol* 17(7):756–65.

73. Spalding MD, Prigge ST (2010) Lipoic acid metabolism in microbial pathogens. *Microbiol Mol Biol Rev* 74(2):200–228.

74. Borziak K, et al. (2014) Comparative genomic analysis reveals 2-oxoacid dehydrogenase complex lipoylation correlation with aerobiosis in archaea. *PLoS One* 9(1). doi:10.1371/journal.pone.0087063.

75. El Zoeiby A, Sanschagrin F, Levesque RC (2002) Structure and function of the Mur enzymes: development of novel inhibitors. *Mol Microbiol* 47(1):1–12.

76. Du W, et al. (2000) Two active forms of UDP-N-acetylglucosamine enolpyruvyl transferase in gram-positive bacteria. *J Bacteriol* 182(15):4146–52.

77. Igarashi K, Kashiwagi K (1999) Polyamine transport in bacteria and yeast. *Biochem J* 344 Pt 3:633–642.

78. Wilks A, Burkhard K a (2007) Heme and virulence: how bacterial pathogens regulate, transport and utilize heme. *Nat Prod Rep* 24:511–522.

79. Hebbeln P, Rodionov D a, Alfandega A, Eitinger T (2007) Biotin uptake in prokaryotes by solute transporters with an optional ATP-binding cassette-containing module. *Proc Natl Acad Sci U S A* 104(12):2909–2914.

80. Fisher DJ, Fernández RE, Adams NE, Maurelli AT (2012) Uptake of Biotin by Chlamydia Spp. through the Use of a Bacterial Transporter (BioY) and a Host-Cell Transporter (SMVT). *PLoS One* 7(9). doi:10.1371/journal.pone.0046052.

81. Morris T, Reed K, Cronan JJ (1995) Lipoic acid metabolism in Escherichia coli: the lplA and lipB genes define redundant pathways for ligation of lipoyl groups to apoprotein. *J Bacteriol* 177(1):1–10.

82. Herbst EJ, Snell EE (1949) Putrescine and related compounds as growth factors for Hemophilus parainfluenzae 7991. *J Biol Chem* 181:47–54.

83. Finkenwirth F, Kirsch F, Eitinger T (2013) A versatile Escherichia coli strain for identification of biotin transporters and for biotin quantification. *Bioengineered* 5(April):1–4.

84. Martens JH, Barg H, Warren MJ, Jahn D (2002) Microbial production of vitamin B12. *Appl Microbiol Biotechnol* 58(3):275–85.

85. Jordan A, Reichard P (1998) Ribonucleotide reductases. *Annu Rev Biochem* 67:71–98.

86. Pfennig N (1978) Rhodocyclus purpureus gen. nov. and sp. nov., a Ring-Shaped, Vitamin B12-Requiring Member of the Family Rhodospirillaceae. *Int J Syst Bacteriol* 28(2):283–288.

87. Lee J, et al. (2009) An alternative polyamine biosynthetic pathway is widespread in bacteria and essential for biofilm formation in Vibrio cholerae. *J Biol Chem* 284(15):9899–907.

88. Pelchovich G, Omer-Bendori S, Gophna U (2013) Menaquinone and iron are essential for complex colony development in Bacillus subtilis. *PLoS One* 8(11):1–14.

89. Nahvi A, Barrick JE, Breaker RR (2004) Coenzyme B12 riboswitches are widespread genetic control elements in prokaryotes. *Nucleic Acids Res* 32(1):143–50.

90. Igarashi K, Kashiwagi K (2010) Characteristics of cellular polyamine transport in prokaryotes and eukaryotes. *Plant Physiol Biochem* 48(7):506–512.

91. Hamana K, Matsuzaki S (1992) Polyamines as a chemotaxonomic marker in bacterial systematics. *Crit Rev Microbiol* 18(4):261–283.

92. Scherer P, Kneifel H (1983) Distribution of polyamines in methanogenic bacteria. *J Bacteriol* 154(3):1315–1322.

93. Layer G, et al. (2010) A novel pathway for the biosynthesis of heme in Archaea: Genome-based bioinformatic predictions and experimental evidence. *Archaea* 2010. doi:10.1155/2010/175050.

94. Collins MD, Jones D (1981) Distribution of isoprenoid quinone structural types in bacteria and their taxonomic implication. *Microbiol Rev* 45(2):316–354.

95. Fujimoto N, Kosaka T, Yamada M (2012) Menaquinone as Well as Ubiquinone as a Crucial Component in the Escherichia coli Respiratory Chain. *Chem Biol*. doi:10.5772/35809.

96. Hiraishi A (1999) Isoprenoid quinones as biomarkers of microbial populations in the environment. *J Biosci Bioeng* 88(5):449–60.

97. Hiratsuka T, et al. (2008) An alternative menaquinone biosynthetic pathway operating in microorganisms. *Science* 321(5896):1670–3.

98. Zhi XY, et al. (2014) The futalosine pathway played an important role in menaquinone biosynthesis during early prokaryote evolution. *Genome Biol Evol* 6:149–160.

99. Streit WR, Entcheva P (2003) Biotin in microbes, the genes involved in its biosynthesis, its biochemical role and perspectives for biotechnological production. *Appl Microbiol Biotechnol* 61(1):21–31.

100. McAllister KA, Peery RB, Zhao G (2006) Acyl carrier protein synthases from gram-negative, gram-positive, and atypical bacterial species: Biochemical and structural properties and physiological implications. *J Bacteriol* 188(13):4737–48.

101. Namboori SC, Graham DE (2008) Acetamido sugar biosynthesis in the euryarchaea. *J Bacteriol* 190(February):2987–2996.

102. Piacente F, et al. (2014) Characterization of a UDP-N-acetylglucosamine biosynthetic pathway encoded by the giant DNA virus Mimivirus. *Glycobiology* 24(1):51–61.

103. Luo H, Lin Y, Gao F, Zhang C-TT, Zhang R (2014) DEG 10, an update of the database of essential genes that includes both protein-coding genes and noncoding genomic elements. *Nucleic Acids Res* 42(November 2013):574–580.

104. Khatiwara A, et al. (2012) Genome scanning for conditionally essential genes in Salmonella enterica Serotype Typhimurium. *Appl Environ Microbiol* 78(9):3098–107.

105. Gallagher LA, Shendure J, Manoil C (2011) Genome-scale identification of resistance functions in Pseudomonas aeruginosa using Tn-seq. *MBio* 2(1):e00315-10.

106. Yang H, et al. (2014) Genome-scale metabolic network validation of Shewanella oneidensis using transposon insertion frequency analysis. *PLoS Comput Biol* 10(9):e1003848.

107. Posnick LM, Samson LD (1999) Influence of S-Adenosylmethionine Pool Size on Spontaneous Mutation, Dam Methylation, and Cell Growth of Escherichia coli. *J Bacteriol* 181(21):6756–6762.

108. Satishchandran C, Taylor JC, Markham GD (1990) Novel Escherichia coli K-12 mutants impaired in S-adenosylmethionine synthesis. *J Bacteriol* 172(8):4489–4496.

109. Driskell LO, Tucker AM, Winkler HH, Wood DO (2005) Rickettsial metK-encoded methionine adenosyltransferase expression in an Escherichia coli metK deletion strain. *J Bacteriol* 187(16):5719–22.

110. Metris A, Reuter M, Gaskin DJH, Baranyi J, van Vliet AHM (2011) In vivo and in silico determination of essential genes of Campylobacter jejuni. *BMC Genomics* 12(1):535.

111. Stahl M, Stintzi A (2011) Identification of essential genes in C. jejuni genome highlights hyper-variable plasticity regions. *Funct Integr Genomics* 11(2):241–57.

112. Asakura H, et al. (2013) Campylobacter jejuni pdxA affects flagellum-mediated motility to alter host colonization. *PLoS One* 8(8):e70418.

113. Scott TA, Hockney RC (1979) Synthesis of vitamin B6 by a mutant of Escherichia coli K12 and the action of 4’-deoxypyridoxine. *J Gen Microbiol* 110(2):285–9.

114. Mah RA, Fung DY, Morse SA (1967) Nutritional requirements of Staphylococcus aureus S-6. *Appl Microbiol* 15(4):866–70.

115. Gretler AC, Mucciolo P, Evans JB, Niven CF (1955) Vitamin nutrition of the staphylococci with special reference to their biotin requirements. *J Bacteriol* 70(1):44–9.

116. Nedenskov P (1994) Nutritional requirements for growth of Helicobacter pylori. *Appl Envir Microbiol* 60(9):3450–3453.

117. Carlsson J (1972) Nutritional requirements of Streptococcus sanguis. *Arch Oral Biol* 17(9):1327–1332.

118. Cummings JE, et al. (2014) The Burkholderia pseudomallei enoyl-acyl carrier protein reductase FabI1 is essential for in vivo growth and is the target of a novel chemotherapeutic with efficacy. *Antimicrob Agents Chemother* 58(2):931–5.

119. Walther B, Karl JP, Booth SL, Boyaval P (2013) Menaquinones, bacteria, and the food supply: the relevance of dairy and fermented food products to vitamin K requirements. *Adv Nutr* 4(4):463–73.

120. Nowicka B, Kruk J (2010) Occurrence, biosynthesis and function of isoprenoid quinones. *Biochim Biophys Acta* 1797(9):1587–605.

121. Yamamoto Y, et al. (2005) Respiration metabolism of Group B Streptococcus is activated by environmental haem and quinone and contributes to virulence. *Mol Microbiol* 56(2):525–34.

122. Brooijmans R, et al. (2009) Heme and menaquinone induced electron transport in lactic acid bacteria. *Microb Cell Fact* 8:28.

123. Kato O, et al. (2010) Quinone-dependent D-lactate dehydrogenase Dld (Cg1027) is essential for growth of Corynebacterium glutamicum on D-lactate. *BMC Microbiol* 10(1):321.

124. Möbius K, et al. (2010) Heme biosynthesis is coupled to electron transport chains for energy generation. *Proc Natl Acad Sci U S A* 107(23):10436–41.

125. Shah P, Swiatlo E (2008) A multifaceted role for polyamines in bacterial pathogens. *Mol Microbiol* 68(1):4–16.

126. Deutscher MP (2009) Maturation and degradation of ribosomal RNA in bacteria. *Prog Mol Biol Transl Sci* 85:369–91.

127. Pramanik J, Keasling JD (1998) Effect of Escherichia coli biomass composition on central metabolic fluxes predicted by a stoichiometric model. *Biotechnol Bioeng* 60(2):230–8.

128. Vu TT, et al. (2012) Genome-scale modeling of light-driven reductant partitioning and carbon fluxes in diazotrophic unicellular cyanobacterium Cyanothece sp. ATCC 51142. *PLoS Comput Biol* 8(4):e1002460.

129. Cotner JB, Makino W, Biddanda BA (2006) Temperature affects stoichiometry and biochemical composition of Escherichia coli. *Microb Ecol* 52(1):26–33.

130. Blazewicz SJ, Barnard RL, Daly RA, Firestone MK (2013) Evaluating rRNA as an indicator of microbial activity in environmental communities: limitations and uses. *ISME J* 7(11):2061–8.
